# An innovative quality improvement approach for rapid improvement of infection prevention and control at health facilities in Sierra Leone

**DOI:** 10.1093/intqhc/mzz137

**Published:** 2020-02-13

**Authors:** Ilka Rondinelli, Gillian Dougherty, Caitlin A Madevu-Matson, Mame Toure, Adewale Akinjeji, Irene Ogongo, Amy Kolwaite, Jamine Weiss, Brigette Gleason, Meghan Marie Lyman, Hassan Benya, Miriam Rabkin

**Affiliations:** 1 ICAP, Columbia University, New York, NY, USA; 2 John Snow, Inc, Boston, MA, USA; 3 ICAP, Country Office, Freetown, Sierra Leone; 4 Division of Healthcare Quality Promotion, Center for Diseases Control (CDC), Atlanta, GA, USA; 5 International Infection Control Program, Center for Diseases Control (CDC), Atlanta, GA, USA; 6 Center for Diseases Control (CDC) Country Office, Freetown, Sierra Leone; 7 Columbia University’s Mailman School of Public Health, New York, NY, USA

**Keywords:** rapid improvement model, quality improvement collaborative, infection prevention and control, Sierra Leone

## Abstract

**Quality challenge:**

The Sierra Leone (SL) Ministry of Health and Sanitation’s National Infection Prevention and Control Unit (NIPCU) launched *National Infection and Prevention Control (IPC) Policy and Guidelines* in 2015, but a 2017 assessment found suboptimal compliance with standards on environmental cleanliness (EC), waste disposal (WD) and personal protective equipment (PPE) use.

**Methods:**

ICAP at Columbia University (ICAP), NIPCU and the Centers for Disease Control and Prevention (CDC) designed and implemented a Rapid Improvement Model (RIM) quality improvement (QI) initiative with a compressed timeframe of 6 months to improve EC, WD and PPE at eight purposively selected health facilities (HFs). Targets were collaboratively developed, and a 37-item checklist was designed to monitor performance. HF teams received QI training and weekly coaching and convened monthly to review progress and exchange best practices. At the final learning session, a “harvest package” of the most effective ideas and tools was developed for use at additional HFs.

**Results:**

The RIM resulted in marked improvement in WD and EC performance and modest improvement in PPE. Aggregate compliance for the 37 indicators increased from 67 to 96% over the course of 4 months, with all HFs showing improvement. Average PPE compliance improved from 85 to 89%, WD from 63 to 99% and EC from 51 to 99%.

**Lessons learned:**

The RIM QIC approach is feasible and effective in SL’s austere health system and led to marked improvement in IPC performance. The best practices are being scaled up and the RIM QIC methodology is being applied to other domains.

## Introduction

Health care-associated infections (HAIs) are a worldwide public health threat, confronting wealthy and poor countries alike and resulting in significant morbidity, mortality and cost [1]. Infection prevention and control (IPC) strategies are critically important to mitigate this global challenge [2] and include a wide range of interventions, including waste management and health facility cleanliness, hand hygiene, appropriate use of personal protective equipment (PPE), injection safety, transmission-based precautions for contact, droplet-borne and airborne infections, HAI surveillance and more [3].

Many IPC interventions are particularly challenging in low- and middle-income countries (LMIC), leading to preventable adverse health outcomes. HAI are two to 20 times more common in LMIC than in high-income countries; for device-associated infections, the risk is 19 times higher. A WHO/UNICEF 2015 global review reported that, globally, nearly 40% of health facilities (HF) lack adequate water, 19% are without sanitation and 35% do not have any hand hygiene materials. As part of the global initiative to achieve universal health coverage, WHO, UNICEF and stakeholders from around the globe committed to the vision that by 2030, every HF in every setting should have safely managed reliable water, sanitation and hygiene facilities and IPC practices that meet standards and patient needs [4].

Sierra Leone is one of the world’s poorest countries, with an estimated per capita income of US$506 and an austere health system [5]. The population of 7.8 million people is served by 1264 HFs, including 24 public hospitals and 16 hospitals owned by a mix of private companies, nongovernmental organizations and faith-based organizations. As of 2016, there were 3 physicians and 50 nurses/midwives for every 100 000 people. Leading causes of death in 2017 were malaria, lower respiratory infections, neonatal disorders and diarrheal disease; life expectancy is 61.4 years for women and 59.5 years for men [6].

The Ebola outbreak of 2014–2015 led to almost 4000 deaths in Sierra Leone and a recognition that IPC services were sorely inadequate. In response, the Ministry of Health and Sanitation (MoHS) established a National Infection Prevention and Control Unit (NIPCU) in 2015 with the support of its development partners, followed by the launch of *National Infection Prevention & Control Policy and Guidelines* in 2015 [7]. MoHS also committed to hiring an IPC Focal Person at every hospital in Sierra Leone, developed a national IPC training curriculum and worked with partners to provide IPC training to HF staff throughout the country.

Despite these interventions, a 2017 national assessment found widespread suboptimal compliance with standards on environmental cleanliness (EC), waste disposal (WD) and use of personal protective equipment (PPE) [8]. In response, ICAP at Columbia University (ICAP) worked with MoHS and the U.S. Centers for Disease Control and Prevention (CDC) to introduce Quality Improvement (QI) methodology to improve IPC in Sierra Leone. ICAP, MoHS and CDC trained staff at four hospitals to conduct systematic root cause analysis, develop practical indicators to monitor performance, identify and prioritize interventions (“change ideas”) and use rapid, iterative testing and learning cycles to find high-impact interventions.

By mid-2017, nurse-led interdisciplinary teams at each of the four hospitals had successfully implemented QI projects to improve hand hygiene, waste management, the appropriate use of PPE and decontamination of surgical instruments. Given the success of these single-HF QI projects, ICAP, MoHS and CDC subsequently designed an innovative variant of the QI Collaborative methodology to rapidly address IPC deficits at scale.

## Methods

Persistent quality challenges in the context of available policies, guidelines, training and staff are characteristic of a “know-do gap” [9], which is often amenable to QI interventions. In particular, the use of Quality Improvement Collaborative (QIC) methodology has successfully led to large-scale health program improvements in low-income settings [10, 11]. The QIC approach, developed by the Institute for Healthcare Improvement, is a well-defined improvement method in which multiple HFs work together to address a common quality challenge, typically over 12–18 months [12].

In QICs, multidisciplinary QI teams are established at each HF, trained and supported to conduct root cause analysis, identify contextually appropriate interventions and conduct rapid iterative tests of change using the Model for Improvement and its plan-do-study-act (PDSA) cycles [13]. All HFs use the same problem statement, targets and indicators, enabling them to compare progress and share interventions and innovations during quarterly learning sessions. Between learning sessions, HF teams receive regular QI coaching visits. At the end of a QIC, a “change package” of best practices, job aides and tools is created which can be used to rapidly disseminate lessons learned [14].

**Figure 1:**
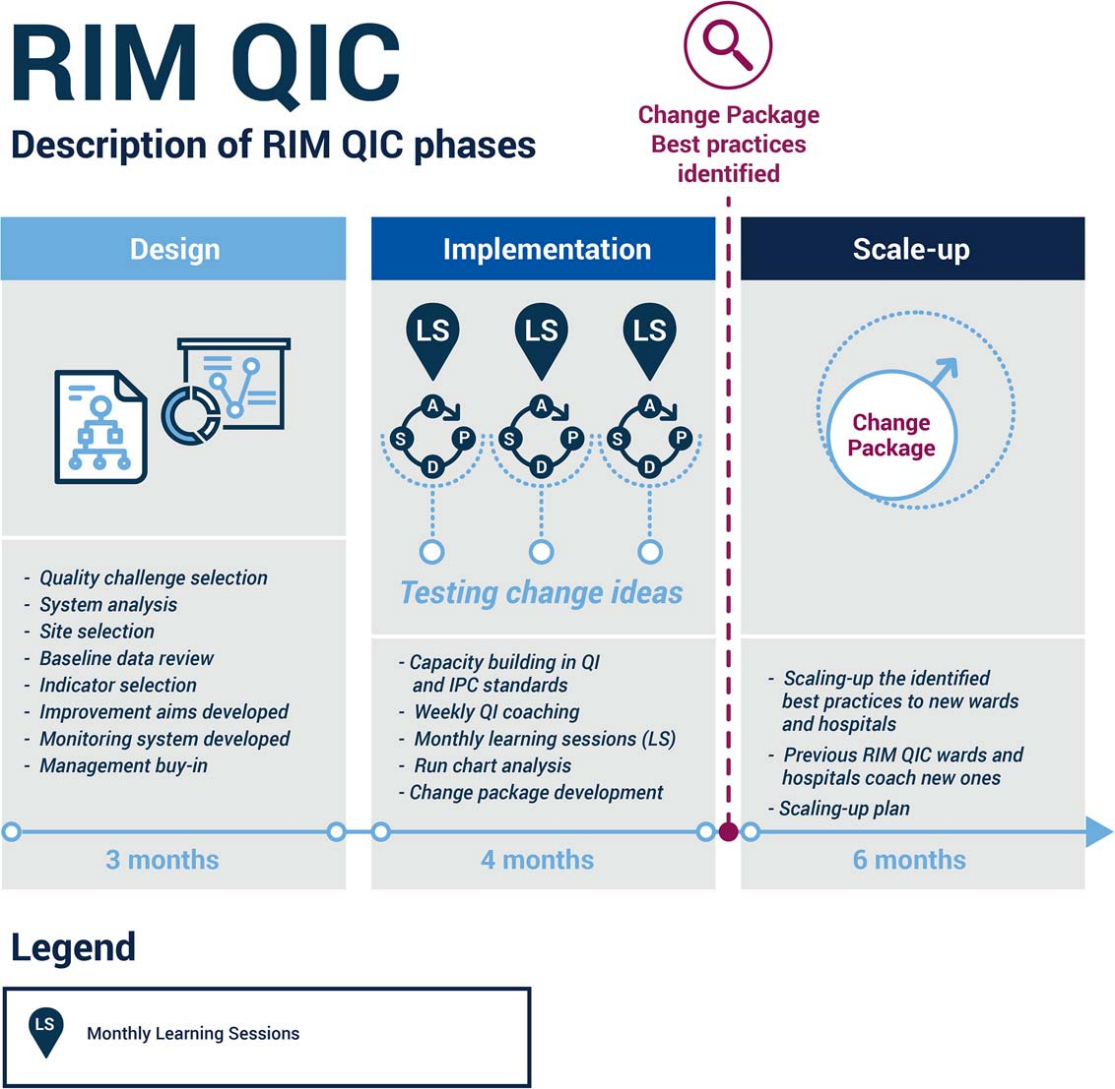
RIM-QIC approach.

In recent years, ICAP has developed an innovative variant on the QIC approach called the “Rapid Improvement Methodology” (RIM). The RIM approach leverages the same strategies as the QIC but compresses activities into a much shorter timeframe (Fig. 1). RIMs use weekly site support visits and monthly learning sessions to complete activities in 3–4 months. Not all quality challenges are amenable to the RIM approach; successful RIM projects require thoughtful selection of quality challenges to ensure that rapid change is possible, a toolkit of pre-existing standardized QI instruments and close follow-up to achieve results.

From November 2017 to April 2018, ICAP, NIPCU and CDC tested the RIM-QIC strategy at eight HFs in Sierra Leone, aiming to improve compliance with national standards related to environmental cleanliness (EC), waste disposal (WD) and the use of PP.

### Design phase: August–October 2017

During the design phase, eight hospitals were purposively selected to participate in the RIM-QIC projects. HF eligibility criteria included the presence of trained IPC teams as well as adequate IPC supplies and infrastructure. Four hospitals that had participated in the earlier “standalone” QI projects were selected, along with four new hospitals. Seven of the HFs were located in the capital city of Freetown and one was in the city of Bo, 108 miles from Freetown.

**Figure 2:**
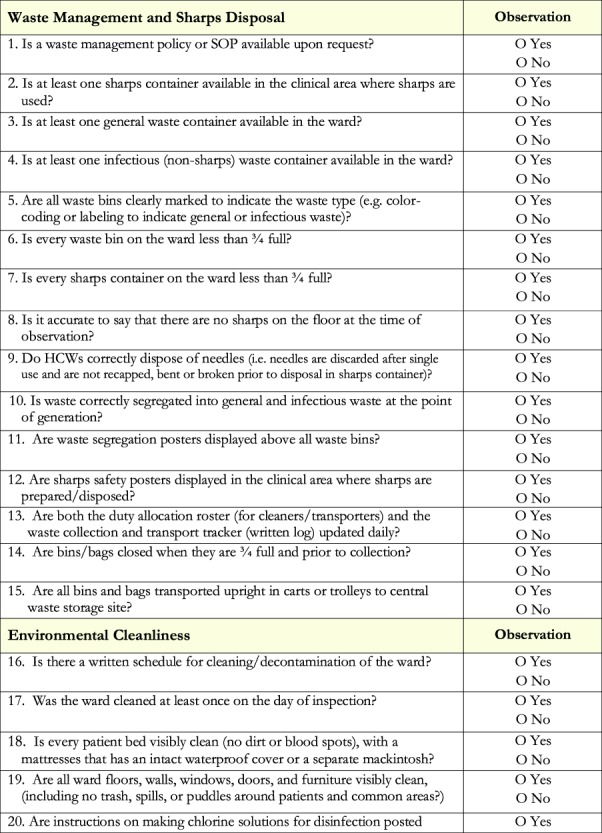

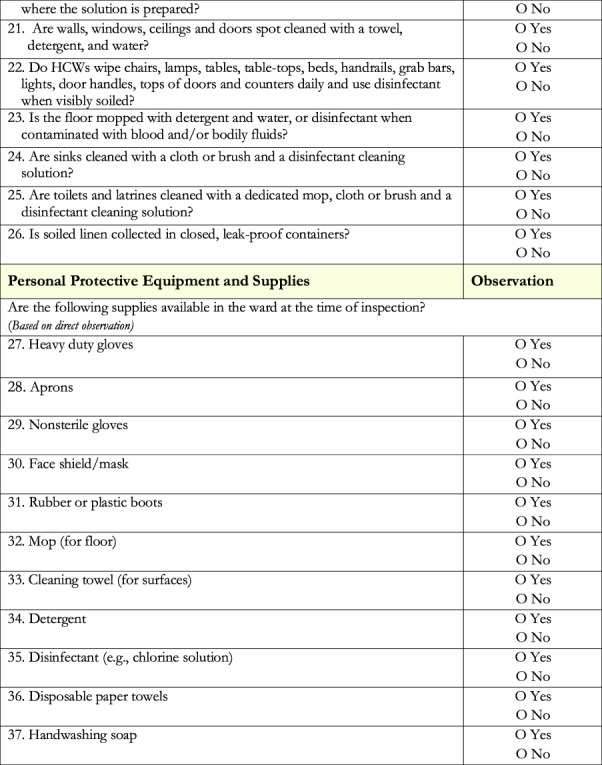
Infection prevention and control quality improvement checklist.

Next, baseline performance in the three prioritized domains was assessed. Existing national IPC WASH M&E indicators were adapted to develop a 37-item observational checklist (Fig. 2), which was then used by ICAP staff to collect weekly data in two wards of each participating HF for 4 weeks. This process revealed that, at baseline, aggregate compliance with the 37 IPC standards at the eight HFs was 67%. Domain-specific compliance was 85% for PPE, 63% for WD and 51% for EC.

With baseline data in hand, ICAP and NIPCU then convened a baseline workshop for 60 participants from the eight HFs to introduce the project, share baseline results and develop shared aim statements and indicators. The sites agreed upon targets of 100% PPE compliance and 90% compliance with WD and EC standards, leading to the following aim statement: *From November 2017 to February 2018, the participating HFs will achieve the following on at least four wards per hospital:*

- Increase in overall compliance to waste management/sharps disposal standards from 63 to 90%;
- Increase in overall compliance to environmental cleanliness standards from 51 to 90%;
- Increase in overall compliance to PPE standards from 85 to 100%.

The project received nonresearch determination from the Columbia University Institutional Review Board (IRB) and approval from MoHS.

### Implementation phase: November 2017–February 2018

Following the baseline workshop, the RIM-QIC was implemented over a period of 4 months, starting with a four-day intensive learning session workshop conducted by ICAP staff to build the QI competencies of HF teams. Content included training on QI methods and the RIM-QIC approach, as well as in-depth discussions about the importance of compliance with IPC standards. The goal of the training was to teach frontline HF staff (nurses, IPC focal persons and IPC ward persons) to apply QI principles and tools to IPC activities at their respective facilities. The participants who attended were the facility QI team members identified during the design phase in collaboration with NIPCU and facility management. The teams represented the four wards in each facility that implemented the IPC RIM-QIC. Each HF team used QI tools to conduct root cause analysis of IPC quality challenges, develop change ideas/interventions for their setting and prioritize change ideas for testing. Teams also familiarized themselves with the composite IPC Checklist and standard operating procedures for data collection.

The ICAP training team designed a robust evaluation of the learning session based on Kirkpatrick’s four levels of evaluation:

The first level, ‘Reaction’, assesses how participants reacted to the training. This was measured using a participants’ survey. Questions included the degree to which participants felt learning objectives had been met, their assessment of the instructors, their opinion of the venue and the degree to which they found modules useful and relevant to their work.

The second level, ‘Learning’, assesses participants’ knowledge acquisition based on their participation in a training. This was measured via pre- and post-tests.

The third level, ‘Behavior’, assesses the degree to which participants apply what they learned from the training when they are back on the job. ICAP assessed this level in the process of supporting site-level.

**Figure 3:**
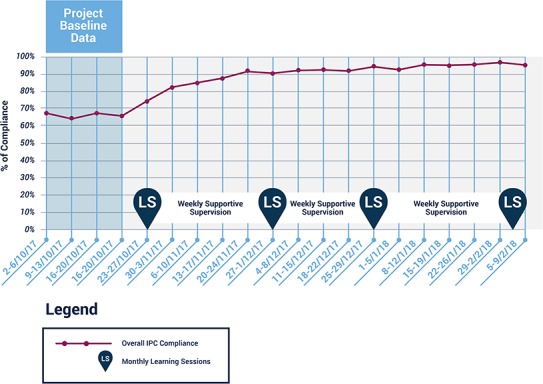
Aggregate compliance for all 37 indicator domains.

The fourth level, ‘Results’, assesses the degree to which targeted outcomes occur. Each facility QI team implemented “change ideas” to improve performance to achieve three aim statements over a period of 4 months, and ICAP monitored and documented the success of these projects.

In addition to gaining knowledge about QI, as evidenced by an average 23% increase in scores on a pre- and post-test, each team left the training with a completed root cause analysis and a priority list of contextually appropriate interventions (“change ideas”) to test using the Plan-Do-Study-Act (PDSA) cycle.

Following the first learning session, ICAP provided intensive support and coaching to each HF QI team. Each team tested a series of prioritized change ideas, collected performance weekly using the RIM checklist and plotted results on an annotated run chart to demonstrate which change ideas led to improvement. Performance data were shared with ICAP using standardized paper forms, and ICAP staff entered the data from each HF into a Microsoft Excel database and a tailored DHIS2 instance that were systematically reviewed on a monthly basis for data quality. If errors were identified, HFs were contacted to obtain correct information. The DHIS2 instance was used to generate weekly descriptive statistics and graphs showing progress toward aim statements for each HF as well as performance of the collaborative as a whole.

ICAP convened monthly learning sessions, at which site-level QI teams from the eight HFs came together for in-depth analysis of their weekly data and run charts. The learning sessions were an important venue for QI teams to share progress and to identify challenges and best practices; they also spurred friendly competition and the dissemination of innovations.

### Harvest phase: March–April 2018

The Harvest Phase included in-depth analysis of the final project data and run charts. At the final workshop (the “harvest session”), HF teams and other stakeholders (including CDC and MoHS/NIPCU) reviewed the change ideas and used set criteria to rank them in order of success. The most successful interventions were documented in a “change package” which grouped them by IPC domain along with specific instructions on how they were implemented.

A final stakeholders’ dissemination meeting was held in April 2018. Thirty-one participants attended the meeting including RIM-QIC team leads and QI team members from all eight HFs as well as representatives from the World Health Organization (WHO), MoHS/NIPCU and CDC. Meeting objectives included informing senior stakeholder leadership of the final project outcomes, reviewing successful change ideas and implementation challenges and developing a plan for spreading the successful change interventions to other wards and HFs.

**Figure 4:**
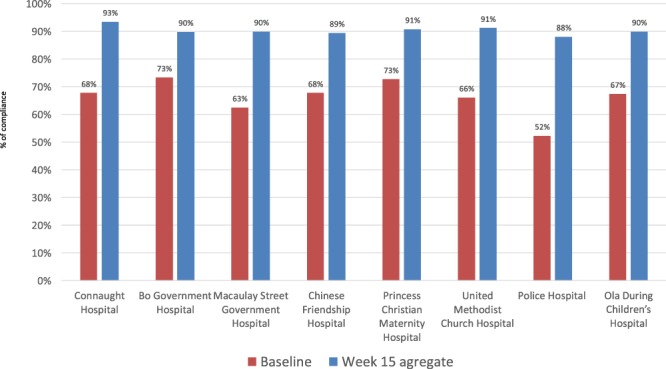
All sites’ overall performance at baseline vs. 15 weeks implementation.

**Figure 5:**
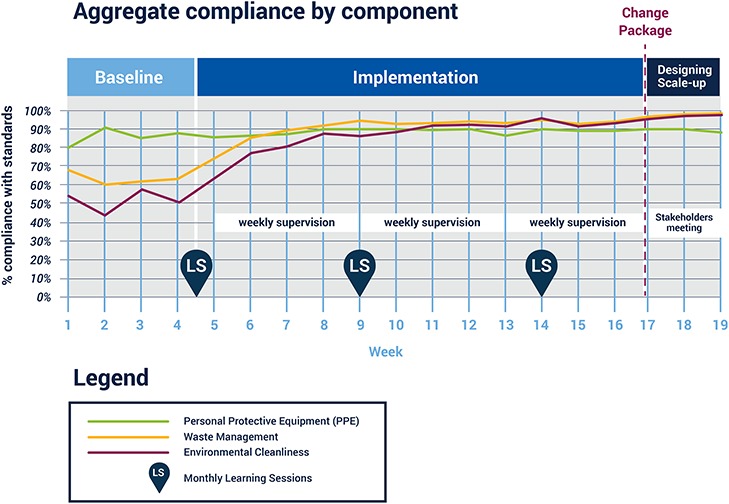
Aggregate performance in three IPC domains.

## Results

All HFs participated in the RIM-QIC throughout the 15-week intervention period, had active QI teams and received supportive supervision and QI coaching as planned. The RIM-QIC learning sessions were well attended, with 53–62 participants attending each of the four learning sessions.

Successful change ideas included refresher IPC trainings, improved job aides to support IPC protocols, more formalized cleaning schedules, improved documentation of waste collection and transport, simulations of IPC challenges, improved inventory, requisition and supply processes and engagement of patients and families.

The RIM-QIC resulted in marked improvement in WD and EC compliance and modest improvement in PPE compliance. Aggregate compliance for all 37 indicators increased from 67 to 96% between week 1 and week 15 (Fig. 3), with all sites showing improvement. The median improvement was 23%, range 16–36% (Fig. 4). In specific domains, PPE improved from 85 to 89%, WD from 63 to 99% and EC from 51 to 99% (Fig. 5); these improvements were sustained for the remainder of the RIM at all HFs. Challenges to improving PPE utilization included stockouts of PPE supplies at some HF during the project.

## Discussion

The RIM-QIC approach led to rapid improvement in IPC performance at the eight HFs, driven largely by improvement in the WD and EC domains. Weekly data collection and analysis ensured ongoing attention to the project, and monthly learning sessions provided a platform for QI team staff to share challenges and offer timely solutions to one another. Sharing results and receiving feedback during monthly learning sessions encouraged staff to overcome challenges and stimulated a sense of friendly competition. Monthly learning sessions also equipped the teams with knowledge, skills and tools to scale up the successful change ideas to additional wards.

Following these improvements at the initial eight HFs, NIPCU expanded the RIM to 11 new HFs without intensive support from ICAP. Outcomes are reportedly excellent and NIPCU plans to scale up the intervention nationwide. Participating HFs have also expanded the RIM-QIC to new wards and other IPC domains without ICAP support. Finally, MoHS has added a QI Unit, which aims to institutionalize the use of QI, and the RIM-QIC approach throughout Sierra Leone’s health system. Based on these experiences, the RIM-QIC approach appears to be feasible, effective and potentially sustainable.

Limitations of the project include its modest scale. Although ICAP has also successfully piloted the RIM-QIC in Angola, this paper presents data that are limited to eight purposively-selected hospitals in Sierra Leone which may not be representative of other HFs in the country or of HFs in other low-income settings. The lack of long-term follow-up data also limits our ability to make conclusions about the sustainability of the intervention.

Strengths of the project include documentation of the novel RIM-QIC approach, a variant on the well-established QIC model, designed by ICAP for specific contexts in which a burst of intensive support can lead to rapid improvement. The approach is most suitable for contexts in which quality challenges can be influenced by site level staff, as opposed to challenges that include “above site” barriers such as policies, staffing levels and procurement of medications, equipment or supplies. RIMs are also facilitated by the availability of pre-existing indicators, agreed-upon standards and targets, and national training curricula. In these contexts, the RIM-QIC strategy may be an optimal approach to bridging the “know-do” gap and accelerating improvements at scale.

## Funding

This work was supported by the US Centers for Disease Control and Prevention (CDC), Global Technical Assistance Project, Cooperative Agreement # 5U2GGH000994-03, under the President’s Emergency Plan for AIDS Relief (PEPFAR).
